# Regulation of Muscle Fiber Type and Running Endurance by PPARδ

**DOI:** 10.1371/journal.pbio.0020294

**Published:** 2004-08-24

**Authors:** Yong-Xu Wang, Chun-Li Zhang, Ruth T Yu, Helen K Cho, Michael C Nelson, Corinne R Bayuga-Ocampo, Jungyeob Ham, Heonjoong Kang, Ronald M Evans

**Affiliations:** **1**Gene Expression Laboratory, Salk InstituteLa Jolla, CaliforniaUnited States of America; **2**Howard Hughes Medical InstituteLa Jolla, CaliforniaUnited States of America; **3**Marine Biotechnology Laboratory, School of Earth and Environmental SciencesSeoul National University, SeoulKorea

## Abstract

Endurance exercise training can promote an adaptive muscle fiber transformation and an increase of mitochondrial biogenesis by triggering scripted changes in gene expression. However, no transcription factor has yet been identified that can direct this process. We describe the engineering of a mouse capable of continuous running of up to twice the distance of a wild-type littermate. This was achieved by targeted expression of an activated form of peroxisome proliferator-activated receptor δ (PPARδ) in skeletal muscle, which induces a switch to form increased numbers of type I muscle fibers. Treatment of wild-type mice with PPARδ agonist elicits a similar type I fiber gene expression profile in muscle. Moreover, these genetically generated fibers confer resistance to obesity with improved metabolic profiles, even in the absence of exercise. These results demonstrate that complex physiologic properties such as fatigue, endurance, and running capacity can be molecularly analyzed and manipulated.

## Abstract 2

Engineered expression of the peroxisome proliferator-activated receptor δ in skeletal muscle increases type I muscle fibers allowing the modified mice to run twice the distance of wild-type littermates

## Introduction

Skeletal muscle fibers are generally classified as type I (oxidative/slow) or type II (glycolytic/fast) fibers. They display marked differences in respect to contraction, metabolism, and susceptibility to fatigue. Type I fibers are mitochondria-rich and mainly use oxidative metabolism for energy production, which provides a stable and long-lasting supply of ATP, and thus are fatigue-resistant. Type II fibers comprise three subtypes, IIa, IIx, and IIb. Type IIb fibers have the lowest levels of mitochondrial content and oxidative enzymes, rely on glycolytic metabolism as a major energy source, and are susceptible to fatigue, while the oxidative and contraction functions of type IIa and IIx lie between type I and IIb (Booth and Thomason 1991; Berchtold et al. 2000; Olson and Williams 2000). Adult skeletal muscle shows plasticity and can undergo conversion between different fiber types in response to exercise training or modulation of motoneuron activity (Booth and Thomason 1991, Jarvis et al. 1996; Pette 1998; Olson and Williams 2000; Hood 2001). This conversion of muscle fiber from type IIb to type IIa and type I is likely to be mediated by a calcium signaling pathway that involves calcineurin, calmodulin-dependent kinase, and the transcriptional cofactor Peroxisome proliferator-activated receptor-gamma coactivator 1α (PGC-1α) (Naya et al. 2000; Olson and Williams 2000; Lin et al. 2002; Wu et al. 2002). However, the targeted transcriptional factors directly responsible for reprogramming the fiber-specific contractile and metabolic genes remain to be identified.

Muscle fiber specification appears to be associated with obesity and diabetes. For instance, rodents that gain the most weight on high-fat diets possess fewer type I fibers (Abou et al. 1992). In obese patients, skeletal muscle has been observed to have reduced oxidative capacity, increased glycolytic capacity, and a decreased percentage of type I fibers (Hickey et al. 1995; Tanner et al. 2002). Similar observations have been made in type 2 diabetic patients (Lillioja et al. 1987; Hickey et al. 1995). Recently, it has been shown that increasing oxidative fibers can lead to improved insulin action and reduced adipocyte size (Luquet et al. 2003; Ryder et al. 2003).

We have previously established that peroxisome proliferator-activated receptor (PPAR) δ is a major transcriptional regulator of fat burning in adipose tissue through activation of enzymes associated with long-chain fatty-acid β-oxidation (Wang et al. 2003). Although PPARδ is the predominant PPAR isoform present in skeletal muscle, its in vivo function has not been determined. Our current study uncovers PPARδ as the first transcription factor able to drive the formation of functional type I muscle fibers, whose activation entrains complex pathways both enhancing physical performance and creating a state of obesity resistance.

## Results

### Activation of PPARδ Leads to Muscle Fiber Transformation

**Figure 1:**
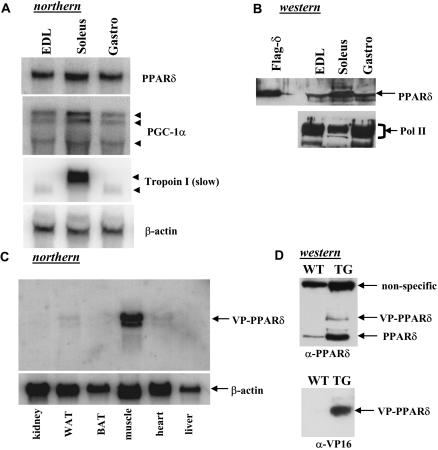
Expression of Endogenous PPARδ and VP16-PPARδ Transgene in Muscle (A) Pooled RNA isolated from various muscles of five wild-type male C57B6 mice was hybridized with indicated probes. EDL, extensor digitorum longus; Gastro, gastrocnemius. (B) Pooled nuclear proteins (15 μg/lane) isolated from muscles of five wild-type male C57B6 were probed with anti-PPARδ antibody. RNA polymerase II (Pol II) is shown as a loading control. (C) Expression of the VP16-PPARδ transgene in various tissues. 10 μg of total RNA from each tissue was hybridized with a VP16 cDNA probe. Gastrocnemius muscle was used here. (D) Nuclear proteins (15 μg/lane) isolated from gastrocnemius muscle of the transgenic mice (TG) and the wild-type littermates (WT) were probed with indicated antibodies. The upper, nonspecific band that cross-reacted with the anti-PPARδ antibody serves a loading control.

A role of PPARδ in muscle fiber was suggested by its enhanced expression—at levels 10-fold and 50-fold greater than PPARα and γ isoforms, respectively (unpublished data). An examination of PPARδ in different muscle fibers reveals a significantly higher level in type I muscle (soleus) relative to type II–rich muscle (extensor digitorum longus) or type I and type II mixed muscle (gastrocnemius) (Figure 1A); this expression pattern closely resembles that of PGC-1α (Lin et al. 2002). A similar pattern but with more pronounced differences was found at the protein level (Figure 1B).

To directly assess the role of activation of PPARδ in control of muscle fiber plasticity and mitochondrial biogenesis, we generated mice expressing a transgene in which the 78-amino-acid VP16 activation domain was fused to the N-terminus of full-length PPARδ, under control of the 2.2-kb human α-skeletal actin promoter. In agreement with the previous characterization of this promoter (Brennan and Hardeman 1993; Clapham et al. 2000), the VP16-PPARδ transgene was selectively expressed in skeletal muscle, with 10-fold less in the heart (Figure 1C). Among different types of muscle fibers, the levels of VP16-PPARδ expression appeared to be similar (unpublished data). As shown in Figure 1D for gastrocnemius muscle, VP16-PPARδ fusion protein was produced at a level similar to that of endogenous PPARδ in wild-type littermates. Interestingly, the level of endogenous muscle PPARδ protein in the transgenic mice was much higher than in the control littermates. The substantial increase of endogenous PPARδ may have been caused by a switch to type I fiber (see below), which intrinsically expresses higher levels of PPARδ (Figure 1A and 1B).

**Figure 2:**
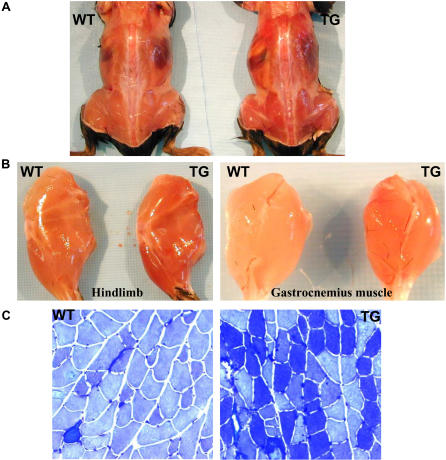
Increased Oxidative Type I Fibers in the Transgenic Mice (A and B) Muscles in transgenic mice (TG) are redder than those in wild-type mice (WT). (C) Metachromatic staining of the type II plantaris muscle. Type I fibers are stained dark blue.

**Figure 3:**
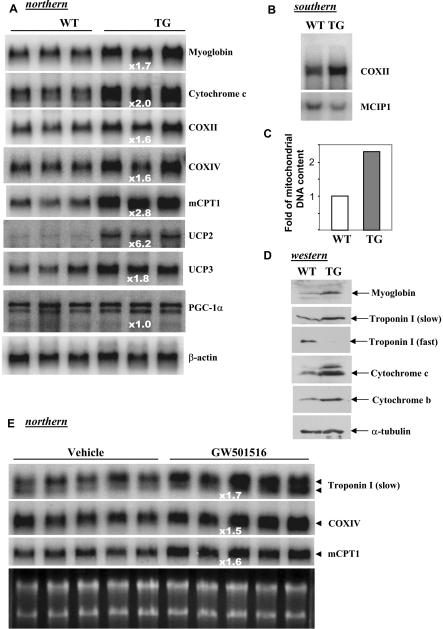
Activation of PPARδ Induces Genes Typical for Type I Fibers and Promotes Mitochondrial Biogenesis (A) Total RNA (10 μg/lane) prepared from gastrocnemius muscle of transgenic (TG) and wild-type (WT) littermates was probed with indicated probes. The fold increase of induction of each gene is shown. (B) Total genomic DNA (10 μg/lane) prepared from gastrocnemius muscle was digested with Nco1 and subjected to Southern analysis with COXII (mitochondrial genome–encoded) and MCIP1 (nuclear genome–encoded) DNA probes. (C) Equal amounts of gastrocnemius muscle were collected from both transgenic mice and control littermates. Total mitochondrial DNA was isolated and separated on 1% agarose gel. The relative abundance of mitochondrial DNA in transgenic and wild-type mice is presented. (D) Western blot analysis of muscle fiber markers and mitochondrial components. Each lane was loaded with 80 μg of total gastrocnemius muscle extracts. (E) Wild-type C57B6 mice were treated with vehicle or PPARδ agonist GW501516 for 10 d. Total RNA (10 μg/lane) prepared from the gastrocnemius muscle was probed with indicated probes.

Type I muscle can be readily distinguished from type II or mixed muscle by its red color, because of its high concentration of myoglobin, a protein typically expressed in oxidative muscle fibers. We found that muscles in the transgenic mice appeared redder (Figure 2A), which is particularly evident in the mixed type I/II fibers of the hindlimb (Figure 2B). Indeed, metachromatic staining revealed a substantial muscle fiber transformation (Figure 2C). In gastrocnemius muscle, we estimated that there was a 2-fold increase of type I fibers. A diagnostic component of oxidative fibers is their high myoglobin and mitochondrial content, which is supported by the mRNA analysis shown in Figure 3A. In addition to myoglobin, mitochondrial components for electron transfer (cytochrome c and cytochrome c oxidase [COX] II and IV) and fatty-acid β-oxidation enzymes were elevated (Figure 3A; unpublished data). These effects appear to be direct consequences of PPARδ activation, as levels of PGC-1α, a coactivator involved in muscle fiber switch and mitochondrial biogenesis (Wu et al. 1999; Lehman et al. 2000; Lin et al. 2002), remained unchanged. Southern blot analysis detected a substantially higher copy number of the mitochondrial genome–encoded COXII DNA in the transgenic mice (Figure 3B). Mitochondrial DNA was increased 2.3-fold in gastrocnemius muscle of the transgenic mice (Figure 3C). These results reveal a marked stimulation of mitochondrial biogenesis and further support the idea that there is a muscle fiber switch. This conclusion was also confirmed by Western blot analysis. As shown in Figure 3D, the characteristic type I fiber proteins, such as myoglobin and cytochrome c and b, were significantly increased. More importantly, the specialized contraction protein troponin I (slow) of type I fiber was robustly induced; this was accompanied by a marked reduction of the specialized contraction protein troponin I (fast) of type II fiber, indicating a high degree of fiber transformation. We next examined whether acute activation of endogenous PPARδ would induce similar target genes. In agreement with the chronic effects in the transgenic mice, we found that, after treatment of wild-type C57B6J mice with the PPARδ-specific agonist GW501516 for only 10 d, genes for slow fiber contractile proteins, mitochondrial biogenesis, and β-oxidation were all upregulated (Figure 3E). This indicates that rapid, systematic, and coordinated changes of muscle fiber properties toward type I can be achieved by activation of the endogenous PPARδ pathway.

### Muscle Fiber Switch by PPARδ Protects Against Obesity

**Figure 4:**
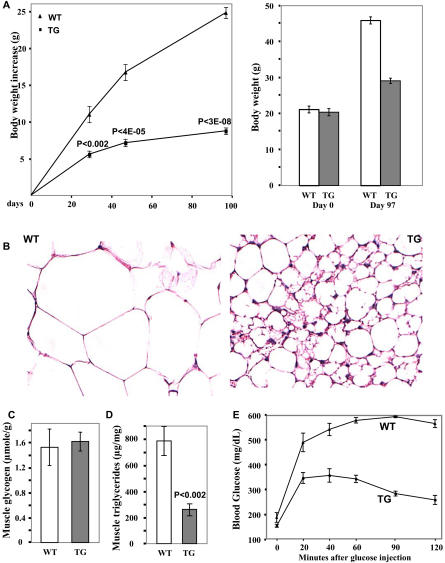
Resistance to High-Fat-Induced Obesity in the Transgenic Mice (A) Seven-week-old transgenic (TG) and wild-type (WT) littermates (*n* = 5–6 for each group) were fed with a high-fat diet for 97 d. Left panel shows net body weight gain, which was calculated for individual mice and then averaged. Right panel shows the body weights before (Day 0) and after (Day 97) high-fat feeding. (B) Histology of inguinal fat pad in the transgenic and wild-type littermates under a high-fat diet for 2 mo. (C and D) Intramuscular glycogen content (C) and triglyceride content (D) of mice in (A) after high-fat feeding (*n* = 6). (E) Glucose tolerance test. Mice in (A) after high-fat feeding were fasted for 6 h and then injected with glucose at a concentration of 1g/kg body weight. Then blood glucose levels were measured periodically over 2 h (*n* = 6).

**Figure 5:**
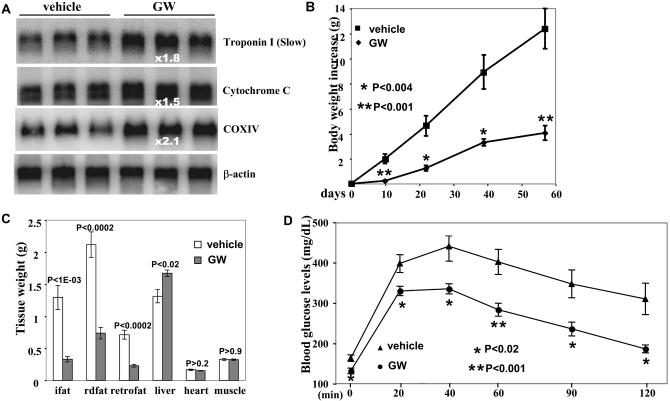
PPARδ Agonists Counteract Obesity Induced by High-Fat Diet (A) Eleven-week-old wild-type C57B6 mice were fed a high-fat diet in combination with vehicle or GW501516 for 57 d. Total RNA (10 μg/lane) prepared from the gastrocnemius muscle was probed with indicated probes. (B) Net body weight gain for mice in (A) after treatment was calculated for individual mice and averaged. Initial body weights were 28.54 ± 1.04 g for vehicle group (*n* = 5) and 28.86 ± 0.80 g for GW501516 group (*n* = 5). (C) Various tissue weights for mice in (A) after treatment. ifat, inguinal fat; rdfat, reproductive fat; retrofat, retroperitoneal fat. (D) Glucose tolerance test. Mice in (A) after treatment were fasted for 6 h and then injected with glucose at a concentration of 1g/kg body weight. Blood glucose levels were then measured periodically over 2 h.

A number of previous studies have shown that obese individuals have fewer oxidative fibers, implying that the presence of oxidative fibers alone may play a part in obesity resistance. To test this possibility, we fed the transgenic mice and their wild-type littermates with a high-fat diet for 97 d. Although the initial body weights of the two groups were very similar, the transgenic mice had gained less than 50% at day 47, and only one-third at day 97, of the weight gained by the wild-type animals (Figure 4A). The transgenic mice displayed significantly higher oxygen consumption on the high-fat diet than the control littermates (unpublished data). By the end of this experiment, the control littermates became obese, whereas the transgenic mice still maintained a normal body weight and fat mass composition (Figure 4A). A histological analysis of inguinal fat pad revealed a much smaller cell size in the transgenic mice (Figure 4B), due to the increased muscle oxidative capacity. While there was no significant difference in intramuscular glycogen content, the triglyceride content was much less in the transgenic mice (Figure 4C and 4D), which may explain their improved glucose tolerance (Figure 4E). We also placed wild-type C57BJ6 mice on the high-fat diet and treated them with either vehicle or the PPARδ agonist GW501516 for 2 mo. GW501516 produced a sustained induction of genes for type I muscle fibers; this, at least in part, resulted in an only 30% gain in body weight, a dramatically reduced fat mass accumulation, and improved glucose tolerance, compared to the vehicle-treated group (Figure 5). Thus, muscle fiber conversion by stimulation with the PPARδ agonist or the activated transgene has a protective role against obesity.

### Activation of PPARδ Enhances Physical Performance

**Figure 6:**
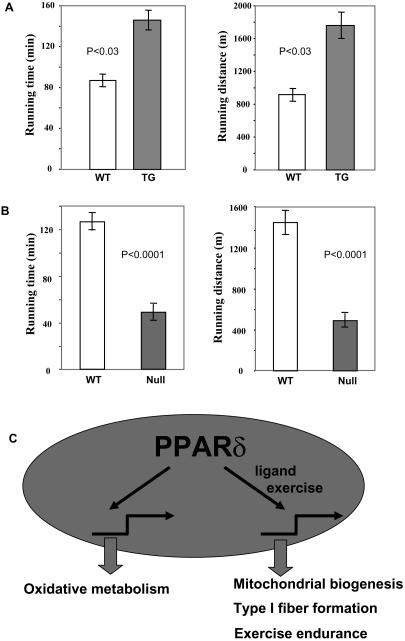
PPARδ Regulates Exercise Endurance (A) Enhanced exercise performance in the transgenic mice. Fourteen-week-old male transgenic and wild-type littermates with similar body weights (*n* = 4 for each group) were subjected to a forced treadmill exercise test. (B) Compromised exercise performance in PPARδ-null mice. Two-month-old male PPARδ-null mice and wild-type controls with similar body weights (*n* = 6 for each group) were subjected to a forced treadmill exercise test. (C) Functions of PPARδ in skeletal muscle.

Muscle oxidative capacity is a crucial factor for determining endurance and fatigue. Indeed, type I fibers adaptively generated through exercise training are considered to be fatigue resistant. However, whether the type I fibers generated molecularly via PPARδ expression can contribute to enhanced performance in the absence of previous training is unclear. In fact, the consequence of genetically induced fiber switch on running capacity has to our knowledge never been evaluated. We thus compared exercise performance between untrained, body-weight-matched transgenic and wild-type littermates. Mice were run on oxygen-infused, enclosed treadmills until exhaustion. Strikingly, the running time and distance the transgenic mice were able to sustain were increased by 67% and 92%, respectively (Figure 6A; also see Videos S1 and S2). The transgenic mice ran about 1 h longer than the controls, which translates to nearly a kilometer further. No significant differences in muscle mass (unpublished data) and daily activity (total counts of activity per hour: 1618 ± 209 for transgenic versus 1987 ± 301 for wild-type, *p* > 0.35, *n* = 4) were observed between the transgenic and control mice. Thus, the remarkable increase in endurance is the physiologic manifestation of muscle fiber transformation. This suggests that genetically directed muscle fiber switch is physiologically and functionally relevant. In addition, we looked at what effect the absence of PPARδ function has on exercise endurance. In the treadmill test, the PPARδ-null mice could sustain only 38% of the running time and 34% of the distance of their age- and weight-matched wild-type counterparts (Figure 6B). These results further support a role for PPARδ in enhancement of physical performance.

## Discussion

Our data reveal that a PPARδ-mediated transcriptional pathway can regulate muscle fiber specification, enabling the generation of a strain of mice with a “long-distance running” phenotype. We show that targeted expression of an activated form of PPARδ produces profound and coordinated increases in oxidation enzymes, mitochondrial biogenesis, and production of specialized type I fiber contractile proteins—the three hallmarks for muscle fiber type switching (Figure 6C). While induction of muscle oxidation enzymes by PPARδ has been seen both in vivo and in vitro (Muoio et al. 2002; Dressel et al. 2003; Luquet et al. 2003; Tanaka et al. 2003; Wang et al. 2003), its effects shown here on muscle fiber switching are unexpected. These progressive changes in oxidative capacity in conjunction with eventual changes in type I muscle fiber lead to a dramatically improved exercise profile and protection against obesity. This does not solely depend on achieving a directed muscle fiber type switch but also requires all the associated changes in neural innervation, motor neuron function, and peripheral metabolic adaptation to enable a new integrated physiological response. Accordingly, activation of muscle PPARδ essentially recapitulates the effects of exercise training even in the absence of training itself. To our knowledge, this has not been directly described for any other transcriptional factor.

The muscle phenotypes described here are remarkably similar to those of transgenic mice expressing either calcineurin, calmodulin-dependent kinase, or PGC-1α (Naya et al. 2000; Lin et al. 2002; Wu et al. 2002), indicating that PPARδ could be one of the hypothetical downstream transcription factors of these pathways. It is important to note that, from our ligand and gain-of-function transgenic studies, PPARδ needs to be activated in order to direct the muscle fiber switch. Indeed, in a recent report by Luquet et al. (2003), simple overexpression of wild-type PPARδ in muscle was found not to be sufficient to promote a fiber switch or obesity resistance, although certain oxidation enzymes were increased. This supports the model in Figure 6C that the activating signal or ligand, but not the receptor, is limiting. Thus, PPARδ activation, rather than merely an increase of PPARδ levels, is an essential element for fiber switching and its associated functional manifestations. How might endogenous PPARδ become activated naturally by exercise training? First, it is possible that exercise generates or increases endogenous ligands for PPARδ as tissues are undergoing substantial increases in fatty-acid internalization and burning. Fatty acids and their metabolites can activate PPARδ. A second model is that exercise may induce expression of PGC-1α (Goto et al. 2000) and thereby activate PPARδ. This is consistent with previous work in which we have shown that PGC-1α physically associates with PPARδ in muscle tissue and can powerfully activate it even in the absence of ligands (Wang et al. 2003). Alternatively, PPARδ may be activated by a distal upstream signaling component such as a kinase cascade. Further dissecting the interactions between PPARδ and its regulatory components will be necessary to fully understand the molecular basis of muscle fiber determination pertinent to exercise training.

Skeletal muscle is a major site to regulate whole-body fatty-acid and glucose metabolism. We show that mice with increased oxidative fibers are resistant to high-fat-induced obesity and glucose intolerance. Moreover, ligand studies provide compelling evidence that activation of endogenous PPARδ can produce similar effects. Might PPARδ have any beneficial effects on glucose metabolism in the lean condition? This has not been explored; however, insulin resistance in the elderly is confined mostly to skeletal muscle and may be due to reduction of mitochondrial number and/or function (Petersen et al. 2003). The ability of PPARδ to stimulate mitochondrial biogenesis and oxidative function suggests that PPARδ could be important for control of insulin resistance during normal aging. Together, these data indicate that PPARδ and its ligands comprise a key molecular switch to regulate muscle fiber specification, obesity resistance, insulin sensitivity, and, most surprisingly, physical endurance. This work demonstrates that complex physiologic properties such as fatigue, endurance, and running capacity can be genetically manipulated.

## Materials and Methods

### 

#### Animals.

The transactivation domain (78 amino acid residues, corresponding to residues 413–490) of VP16 was fused in frame with the N-terminus of mouse PPARδ. The VP16-PPARδ fusion cDNA was placed downstream of the human α-skeletal actin promoter (Brennan and Hardeman 1993), and upstream of the SV40 intron/poly(A) sequence. The transgene was purified and injected into C57BL/6J × CBA F1 zygotes. Transgenic mice were backcrossed with C57BL/6J for two generations. Wild-type littermates were used as controls throughout the study. On normal chow diet, the transgenic mice and control littermates used here had similar body weights. PPARδ-null mice were previously generated (Barak et al. 2002). Mice were fed either a standard chow with 4% (w/w) fat content (Harlan Teklad, Harlan, Indianapolis, Indiana, United States) or a high-fat diet containing 35% (w/w) fat content (product F3282, Bioserv, Frenchtown, New Jersey, United States) as indicated. For ligand experiments, we synthesized the GW501516 compound and mice were orally gavaged daily (10 mg/kg or vehicle alone).

#### Gene expression analysis and physiological studies

Mouse EST clones were obtained from ATCC (Manassas, Virginia, United States), verified by sequencing, and used as Northern probes. Antibodies were obtained from Santa Cruz Biotechnology (Santa Cruz, California, United States). Total muscle protein extracts (Lin et al. 2002) and nuclear proteins (Wang et al. 2003) were prepared as described.

Prior to the exercise performance test, the mice were accustomed to the treadmill (Columbus Instruments, Columbus, Ohio, United States) with a 5-min run at 7 m/min once per day for 2 d. The exercise test regimen was 10 m/min for the first 60 min, followed by 1 m/min increment increases at 15-min intervals. Exhaustion was defined when mice were unable to avoid repetitive electrical shocks.

#### Muscle fiber typing and mitochondrial DNA isolation

Muscle fiber typing was essentially performed using metachromatic dye–ATPase methods as described (Ogilvie and Feeback 1990). Muscle mitochondria were isolated (Scholte et al. 1997). Mitochondrial DNA was prepared and analyzed on 1% agarose gel.

#### Statistical analysis

Number of mice for each group used in experiments is indicated in figure legends. Values are presented as mean ± SEM. A two-tailed Student's t test was used to calculate *p-*values.

## Supporting Information

Video S1 Beginning of Running Test This video shows the exercise performance of a representative of the transgenic mice (right chamber) and a representative of wild-type control littermates (left chamber) on the treadmill 15 min into the exercise challenge.
(52.4 MB MOV).

Video S2 Running Test 90 Min Later This video shows the exercise performance of a representative of the transgenic mice (right chamber) and a representative of wild-type control littermates (left chamber) on the treadmill 90 min into the exercise challenge.
(41.7 MB MOV).

## Abbreviations

COX: cytochrome c oxidase
mCPT1: muscle carnitine palmitoyltransferase-1
PGC-1α: Peroxisome proliferator-activated receptor-gamma coactivator 1α
PPAR: peroxisome proliferator-activated receptor
UCP: uncoupling protein
