# Correction: Regulation of Muscle Fiber Type and Running Endurance by PPARδ

**DOI:** 10.1371/journal.pbio.0030061

**Published:** 2005-01-18

In ***PLoS Biology***, volume 2, issue 10:

Regulation of Muscle Fiber Type and Running Endurance by PPARδ.

**Wang YX, Zhang CL, Yu RT, Cho HK, Nelson MC, et al.**

10.1371/journal.pbio.0020294

The following competing interest should have been indicated in the above research article. R. M. Evans wishes to acknowledge a consulting relationship with Ligand Pharmaceuticals. Under a licensing agreement with GlaxoSmithKline, Ligand receives milestone payments on the development of GW501516, a PPARδ-specific drug used in this study. For clarification, no materials or support were received from either company, and no agreements were in place concerning the execution or publication of this work.

Published January 18, 2005

